# Full Quivers

**Published:** 1889-01-19

**Authors:** 


### BABIES.

(Continued from p. 239.)

### III.—FULL QUIVERS.

THE advent of a baby—"a living, crying, and aggressive fact," as somebody calls it—is a great event in any household. Well may John Chinaman pray, as he is said to do on such occasions, that the child may be easy to take care of, and that it may sleep well o' nights. It is a great event. It topsy-turveyizes the house, turns night into day, hurls papa from the proud pinnacle of his autocracy, and sets up in his place strange powers and potentates who barricade him out of his own quarters, reduce him to the status of a lodger in his own house, and assume all but unlimited control of his time, his attention, and his purse. Under such circumstances it must often occur to the ill-used master of the house that there is really a good deal to be said in justification of the custom, said to prevail among some Orientals, for papa to go to bed on the birth of a child, and for visits of condolence and sympathy to be paid to him.

It is a great event, the arrival of a baby. But the arrival of two babies!—two "living, crying, and aggressive facts!" What an event is that. How much more fervently might the Chinaman pray that they may be easy to take care of, and may sleep well o' nights! If the paternal visage could be always photographed at the moment the tidings of his exceptional fortune are communicated, what an interesting collection of physiognomies could be selected from such photographs! And if the paternal exclamations and remarks—sacred and profane—could all be accurately reported, what an interesting volume they would afford! As for those more highly distinguished individuals who have three, four, five, or six suddenly added to their establishment, they would probably afford nothing of exceptional interest, either in word or facial expression. When a man gets beyond two, he must feel that a few more or less cannot very much matter, and whether the good lady stops at number three or goes on to three hundred and sixty-five, as the Countess of Henneberg is said to have done in 1276, neither voice nor face can at all adequately express the sense of paternity in such a case. That record of the Countess of Henneberg, by the way, perhaps is not implicitly to be relied on. The story goes that she applied an opprobrious epithet to a beggar woman merely because she had borne twins. Incensed at this the woman prayed that her ladyship might have as many children at a birth as there are days in the year, and on the following Good Friday the prayer was answered. It is not necessary that this old story shall be implicitly believed, although at one time of day it seems to have been very generally credited. "By waggon to Lausdune, where the 365 children were born," writes Samuel Pepys in his famous Diary under the date May 19th, 1660. "We saw the hill where they say the house stood wherein the children were born. The basins wherein the male and female children were baptized do stand over a large table that hangs upon a wall with the old story of the thing in Dutch-Latin, beginning 'Margarita Herman Comitissa,' &c. The thing was done about two hundred years ago." Clearly Samuel Pepys was not prepared to say such a thing could not have happened. But most people may find their credulity quite sufficiently taxed by a more recent event, said to have taken place in Italy, where a woman, now living, is affirmed, seemingly on respectable authority, to have presented her husband with no less than six boys at once. This may be taken to be the greatest achievement of the kind recorded in modern times, and the next best is accredited to the same woman. A very remarkable family career has been this Italian peasant's, if the confident assertions of the Italian newspapers may be relied upon. They have declared that it is beyond reasonable question that she has a living family of 62 children, and that they have arrived, some of them singly, others in little groups of from two to six; that in one case there were four, and in another five. There have been papas more blessed than even the good man of this little Italian household. The King of Siam, for instance—who is under 40 years of age, and may yet live to know much more of the responsibilities of paternity—is said to have 263 children. But then, of course, the King of Siam is more married than most men. For a married couple the Italian peasant Granata and his wife can probably challenge comparison with the greatest of their race—assuming, of course, that some of the best Italian newspapers were not egregiously hoaxed in this matter—though it is possible that there may be parts of the world in which they would be considerably less distinguished than in Italy.

Nations differ very greatly with respect to the number constituting the family party. North American Indians are said to average only two or three to a family; and five children are considered to be quite a large one, notwithstanding that it is the custom for their young people to set up the wigwam at twelve or fifteen years of age. The Fuegians and Australian aborigines have very low powers of multiplication, and the Hottentot family rarely exceeds two or three. On the other hand, the Boers and Kaffirs are exceedingly prolific. For little Kaffirs to come in braces has been said to be almost as common as for them to come singly, and triplets are not infrequent. So at least it has been recorded in Mr. Herbert Spencer's "Principles of Biology." In the same work it is said that the French Canadians, home-loving, contented, easy-going, and prosperous as they usually have been, are blessed with very full quivers; *nous sommes terrible pour les enfants*, said one of them. Eight to sixteen their families commonly number, and the authority quoted for this statement says that he knew one or two women who have had twenty-five and threatened *le vingt sixième pour le prêtre*.

At one extremity we have the North American Indians, or the Aboriginal Australians; at the other may perhaps be placed the Brazilians. Walsh, who some years ago wrote some very intelligent observations on that vast-tropical land, says that the Brazilian mothers are exceedingly prolific. They marry, it appears, at twelve or thirteen, and they continue to have children to a late period in life. Nevertheless, they appear to be as careful to improve each shining hour as though their time was exceptionally limited. Families, says this authority, often run up to an incredible number. He gives instances in illustration of this, and mentions one woman who came to church with four infants to be baptized, and declares that similar cases were to be met with on all hands. The queerest little family he met with in Brazil was one of three infants all the same age, but one of them a white, another a black, and a third a creole.

There is something very dismaying in the idea of a sudden addition of four or five babies to a household; but there is nothing at all incredible, even in such a case as that reported from Italy. The cases of twins occurring in this country are computed to be about one in a hundred births, and although an ancient philosopher declared five to be the limit of human fecundity, it may perhaps be considered very probable that he was well within the mark, when it is remembered that in the United Kingdom alone more than a hundred cases of triplets are annually brought under the attention of the Queen for the purpose of obtaining the Royal bounty. It is quite certain that there are many others besides these, since, of course, it is only those parents who are in needy circumstances who make the application, and, indeed, not all of them. It is easy to believe, therefore, that not infrequently in various odd corners of the earth, little surprises for expectant papas and mammas do come quite up to our old philosopher's high-water mark, and may occasionally go beyond it. It is beyond question that four births at a time have occasionally occurred in this country, and even five have been reported. Haller ventured on the assertion that quadruplets were born once in 20,000 times, while five, he said, were not known once in a million times. One such occurrence may be regarded as beyond dispute, since the bodies of the children have been preserved in the Hunterian Museum. This phenomenon took place at Blackburn in 1786, two of the little people being born dead, and the other three dying after a short time. There seems to be no reliable evidence of any other similar occurrence in this country, and an eminent medical man at the time this took place, said, in a letter addressed to Sir Joseph Banks, the president of the Royal Society, that he had requested various friends in St. Petersburg, Berlin, and Vienna, Lyons, Paris, and Ghent to search for similar well-authenticated instances of the kind, but that they had been unable to find any. Fifty years or so previously, however, there appear to have been two cases in one year on the Continent, one near Prague, in Bohemia, the other in Upper Saxony; while, if an occurrence of multiple birth, mentioned once before the Royal Society, may be accepted as reliable, the mother of the noble house of Valdemeure had in the first year of marriage two children, then three, four, and five, and in the fifth year six children at a birth. Some support is given to this story by the fact that at the time it was publicly recorded by Ambrose Pére, one of the six was asserted to be living, and the Lord of Valdemeure. The mother died in her last confinement, and it seems hardly probable that such a statement with regard to a living nobleman would have been made if it had not been true. It is not more remarkable than the similar occurrence lately reported from Italy, and though the regular year by year increase may be thought a suspicious circumstance, it is probably just one of those circumstances which would not have been invented, and perhaps ought to be regarded as confirming the truth of the statement. It is allowed on all hands that one distinguished achievement in this way is very apt to be followed by another.

*(To be continued.)*

